# The Metabolism of the Enamel in Relation to the Resistance of Caries

**Published:** 1912-10-15

**Authors:** Theo. Von Beust

**Affiliations:** Dresden


### THE METABOLISM OF THE ENAMEL IN RELATION TO THE RESISTANCE OF CARIES.

BY DR. THEO. VON BEUST, DRESDEN.

All our attempts to get at the truth of this matter of the resistance of caries are wrecked upon the question as to whether the enamel has very little metabolic power or none at all. My aim here is to demonstrate that defensive measures are, even in the case of the enamel, not by any means excluded.

Let us compare the impression we obtain from "healed" caries with the result of the following experiment. If we take a sound tooth, one that has been lying for some time in alcohol, and put it into the cork of a bottle in the way so that the apical end of the root is soaked in a solution of aniline red in alcohol to which a good pinch of sodium chloride has been added in order to set going the diffusion streams, we shall find that during a period varying from two to eight days the part of the enamel lying nearest to the pulp has taken on a reddish coloring. If suitable sections of such teeth are strongly magnified, it becomes clearly evident that the solution has penetrated into the enamel. I will deal a little later with Caush's statements in the *Dental Cosmos* and also with the question of the exact working of this process. For the present I should like to point out that the so-called "spindles" are none other than Tome's fibrils, proceeding, now tuft-wise, now in solid cord-like formation into the enamel and breaking up there into fine branchlets. They cannot for a moment be considered as anomalies nor may they be called non-calcified cement substance. They are canals which lie, my research up to the present time leads me to conclude, not in but between the prisms of the enamel. They are related to the interprismatic cortical substance in so far as it is colored by this method, for only through these canals could the colored solution reach this substance. It is often possible to follow these vessels, perfectly filled with red-colored organic substance, right up to the margin of the enamel. Much of the enamel is highly vascular. A metabolism which is not without some significance, and taking place by means of the vascular system of the pulp, stands now clear of any doubt. The canals can sometimes be traced into Nasmyth's membrane.

If we consider these observations in relation to a progressive caries we must admit the possibility of an obstruction of the process; for if we compare the slow progress of caries with the relatively slight accessibility of the enamel to tissue-lymph we cannot but judge that the metabolism is sufficient.

The advance of the caries is so slow, the point of the aggressive cone so very minute, that we can accept the presence of a means of defense in the organism of the enamel with as little difficulty as in the case of other tissues. Though this discussion is intended to bear a purely provisional character, I should like nevertheless to bring forward a few clinical considerations related to its general purport.

1. Is the consistency of the enamel in a young tooth different from that of an adult?

2. Does the appearance of the teeth in one patient vary at all strikingly at definite periods?

3. Does the enamel of teeth where the pulp has died early, remain weak and brittle?

4. Is the enamel of a dead tooth drier than that of a living one?

5. Does the enamel as a whole take on color from within outwards—for instance, in the case of copper-amalgam filling?

6. Can there be found on almost any tooth, including the teeth of the "immune," marks of caries when the disease has healed?

The foregoing remarks may indeed bear further interpretation, but in any case they support my opinion as to an exchange of lymphs in the tooth.

On no account must these experiments be understood as furnishing any explanation of susceptibility to caries. The resistance to caries, as to other stimuli, is relative. This does not, however, interfere with my suggested conclusion that the proven metabolism is the defensive agent. Equally, on the other hand, must we abstain from accepting this process as the sole protection of the enamel. The lymph in the mouth, as it is found in the saliva, is in this connection certainly not to be ignored.—(Abstracted from a reprint in *The Dental Record.*

